# Bilateral hydronephrosis due to obstructive ureteral stone associated with norovirus gastroenteritis

**DOI:** 10.1002/ccr3.952

**Published:** 2017-05-02

**Authors:** Fumihiro Ochi, Kenji Furuno, Pin Fee Chong, Junichiro Tezuka, Yumi Mizuno, Tomonobu Aoki, Eiichi Ishii

**Affiliations:** ^1^ Department of PediatricsEhime University Graduate School of MedicineToonEhimeJapan; ^2^ Department of Pediatric Infectious DiseasesFukuoka Children's HospitalFukuokaFukuokaJapan; ^3^ Department of General Pediatrics and Interdisciplinary MedicineFukuoka Children's HospitalFukuokaFukuokaJapan; ^4^ Department of Allergy and Respiratory MedicineFukuoka Children's HospitalFukuokaFukuokaJapan

**Keywords:** Gastroenteritis, hydronephrosis, norovirus, ureteral calculi, uric acid

## Abstract

Recently, cases of urinary tract calculi causing hydronephrosis and postrenal renal failure associated with viral gastroenteritis were documented, yet few were related to norovirus. During norovirus gastroenteritis, observation of oliguria, aciduria, low FENa value, and elevation of blood or urinary uric acid level may necessitate clinical workout for nephrolithiasis.

## Introduction

In most children, renal stones are associated with a urinary metabolic abnormality, urinary tract infection, and/or a structural renal or urinary tract abnormality 1, 2, 3, 4. In addition, some patients with acute obstructive uropathy due to ammonium acid urate (AAU) stones, developing approximately 6–7 days after the onset of rotavirus gastroenteritis, have been reported, mainly in Japan 4.

Cases of urinary tract calculi that cause hydronephrosis and postrenal failure associated with viral gastroenteritis have been recently documented; however, few cases were related to norovirus 5, 6.

## Case Report

Here, we report the case of a 4‐year‐old girl with bilateral hydronephrosis because of obstructive ureteral stone associated with norovirus gastroenteritis. She had been healthy with no previous medical problems. No family history of urolithiasis was noted. She had frequent vomiting 5 days before hospitalization and complained of abdominal pain 3 days later. Stool testing for norovirus antigen (QuickNavi™‐Norovirus2, Denka Seiken Co., Ltd Tokyo, JAPAN.) was positive; thus, norovirus gastroenteritis was diagnosed. Dysuria developed 1 day before hospitalization. Laboratory examination revealed the following: hyperuricemia, 10.8 mg/dL; blood urea nitrogen (BUN), 20 mg/dL; and serum creatinine (Cre), 0.25 mg/dL.

On admission, bilateral costovertebral angle and lower abdominal tenderness were observed. Laboratory results were as follows: white blood cell count, 8520/*μ*L; BUN, 21 mg/dL; serum Cre, 0.27 mg/dL; uric acid (UA), 4.9 mg/dL; sodium, 135 mEq/L; potassium, 4.3 mEq/L; and chloride, 97 mEq/L. Venous blood gas examination demonstrated blood pH of 7.394; HCO_3_^−^, 21.0 mmol/L; and base excess, −3.2 mmol/L.

**Figure 1:**
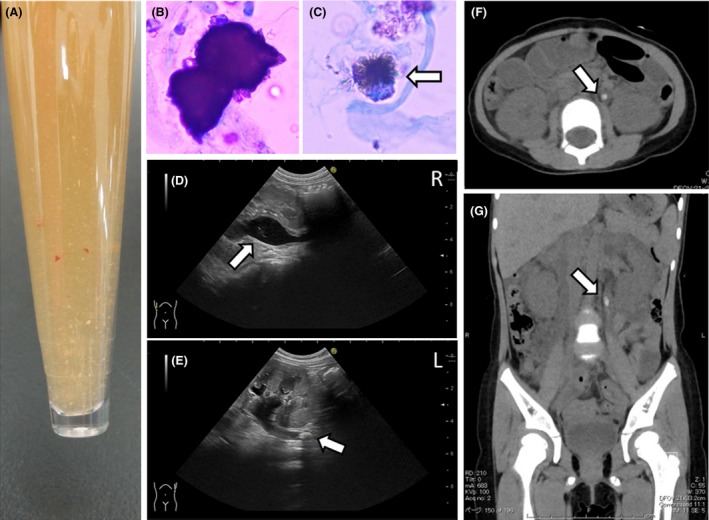
(A) Urine appearance. White and brown fine sandy stones were observed in urine. (B) Ammonium acid urate crystal with haw‐like appearance in the urinary sediment (Sternheimer stain). (C) Sodium urate crystal in the urinary sediment (Sternheimer stain) with spinal‐like appearance (arrows). (D, E) Abdominal ultrasound revealing dilated bilateral pelvises and calculi in the left ureter on admission. Arrows show some fine high echoic lesions in bilateral pelvises. (F, G) Abdominal computed tomography showing dilated calculi in the left ureter on admission. (F) Axial section and (G) coronal section. Arrows indicate urinary stone (4.2 × 8.7 mm) in the left upper ureter.

There was spontaneous excretion of white and brown fine sandy stones in her urine (Fig. 1A). Urinalysis showed hematuria, aciduria, and ketonuria, with AAU and sodium urate crystals (Fig. 1B and C). The urinary UA level was 70.6 mg/dL, and the fractional excretion of sodium (FENa) level was 0.043%. Stool tests for rotavirus and adenovirus antigen and urinary culture were negative.

Urinary ultrasonography and abdominal computed tomography (CT) showed bilateral hydronephrosis (grade 2) with renal cortical thinning, bilateral renal pelvis calculi, and left upper ureteral calculus (Fig. 1D–G). Intravenous fluid therapy was administered, and spontaneous passage of calculi occurred with subsequent hydronephrosis resolution. The patient was discharged, and follow‐up CT and ultrasonography demonstrated the absence of bilateral hydronephrosis and ureteral calculus.

## Discussion

Urinary calculi should be recognized as an important complication of acute gastroenteritis. They cause acute abdomen but may be overlooked, resulting in late diagnosis of acute gastroenteritis because of similar clinical symptoms. Persistence of oliguria despite apparent recovery of gastrointestinal symptoms should prompt clinicians to consider nephrolithiasis.

Major components of viral gastroenteritis‐associated urinary tract calculi were from the urinary acid system, particularly AAU 7. AAU or urate stones, usually observed in gastroenteritis, cannot be detected by kidney–ureter–bladder X‐ray. Urinary stone and bilateral hydronephrosis can be diagnosed by ultrasonography and abdominal CT.

The formation mechanism of AAU and urate stones remains unclear. Dick et al. described the pathophysiological mechanism of AAU calculi formation in patients with laxative abuse 8. Yokoyama et al. proposed the mechanism for the formation of urate or AAU crystals and calculi in rotavirus gastroenteritis 9. Both studies theorized that gastrointestinal loss of water and electrolytes, such as sodium, causes metabolic acidosis, aciduria, ketonuria, increased urinary ammonium ion concentration, and decreased urine volume. Metabolic acidosis may lead to acidification of urine, with subsequent UA crystal formation. Furthermore, hyperuricemia associated with rotavirus or norovirus gastroenteritis has been reported 5, 6. Low urinary sodium levels promote coupling of urate with abundant ammonium ions. Yokoyama et al. reported that FENa was extremely low in the AAU crystal group compared with that in the no‐crystal group 9. Our patient had extremely low FENa levels and severe hyperuricemia.

As in our case, hyperuremia, aciduria, and dehydration developed in the early stage of norovirus gastroenteritis, which may have precipitated urine UA supersaturation and decreased urinary UA solubility, resulting in the formation of AAU stones 5–6 days after norovirus infection onset.

Thus, observation of oliguria, aciduria, low FENa level, and elevated blood or urinary UA level may necessitate examination for nephrolithiasis during norovirus gastroenteritis.

## Authorship

FO, KF, PFC, JT, YM, and TA: managed the patient and prepared the manuscript. FO, KF, PFC, and EI: reviewed the manuscript. All authors: read and approved the final manuscript.

## Conflict of Interest

None declared.
